# Design and Evaluation of pH Sensitive PEG-Protamine Nanocomplex of Doxorubicin for Treatment of Breast Cancer

**DOI:** 10.3390/polym14122403

**Published:** 2022-06-14

**Authors:** Ikhlaque Ahmad, Muhammad Farhan Ali Khan, Abbas Rahdar, Saddam Hussain, Fahad Khan Tareen, Muhammad Waqas Salim, Narges Ajalli, Muhammad Imran Amirzada, Ahmad Khan

**Affiliations:** 1 Department of Pharmacy, Faculty of Biological Sciences, Quaid-i-Azam University, Islamabad 45320, Pakistan; ikahqau@gmail.com (I.A.); farhanali@bs.qau.edu.pk (M.F.A.K.); pharmacistsaddam@gmail.com (S.H.); smws006@gmail.com (M.W.S.); 2 Department of Physics, Faculty of Science, University of Zabol, Zabol 98613-35856, Iran; 3 Faculty of Pharmacy, Capital University of Science and Technology, Islamabad Expressway, Kahuta Road, Zone-V, Islamabad 45320, Pakistan; fahadkhantareen@hotmail.com; 4 Department of Chemical Engineering, Faculty of Engineering, University of Tehran, Tehran 98613-35859, Iran; narges.ajalli@ut.ac.ir; 5 Department of Pharmacy, COMSATS University Islamabad, Abbottabad Campus, Abbottabad 22010, Pakistan

**Keywords:** breast cancer, nanoparticles, doxorubicin, PEG-protamine complex

## Abstract

Cancer is the most common cause of mortality worldwide. There is dire need of modern strategies—such as surface modification of nanocarriers—to combat this global illness. Incorporation of active targeting ligands has arisen as a novel platform for specific tumor targeting. The aim of the current study was to formulate PEG-protamine complex (PPC) of doxorubicin (DOX) for treatment of breast cancer (BC). DOX coupling with PEG can enhance cell-penetrating ability: combating resistance in MDA-MB 231 breast cancer cells. Ionic gelation method was adopted to fabricate a pH sensitive nanocomplex. The optimized nanoformulation was characterized for its particle diameter, zeta potential, surface morphology, entrapment efficiency, crystallinity, and molecular interaction. In vitro assay was executed to gauge the release potential of nanoformulation. The mean particle size, zeta potential, and polydispersity index (PDI) of the optimized nanoparticles were observed to be 212 nm, 15.2 mV, and 0.264, respectively. Crystallinity studies and Fourier transform infrared (FTIR) analysis revealed no molecular interaction and confirmed the amorphous nature of drug within nanoparticles. The in vitro release data indicate sustained drug release at pH 4.8, which is intracellular pH of breast cancer cells, as compared to the drug solution. PPC loaded with doxorubicin can be utilized as an alternative and effective approach for specific targeting of breast cancer.

## 1. Introduction

Cancer is the most common cause of mortality worldwide. Breast cancer is the second-most reported cancer with distribution of 1.38 million and 10.9% of total, becoming a major cause of morbidity and mortality among women in 2008. The mortality rate with breast cancer is higher in lower income countries which approximately reaches up to 60%. The fourth-most common and widely distributed cancer is colorectal cancer with distribution of 1.23 million and made 9.7% of total population [1,2].

Cancers (especially breast cancers) can be classified in two ways: areas affected and the level of damage. Those two arches of cancer are termed ‘carcinomas’ and ‘sarcomas’. The cancers or outgrowths that are seen in the epithelial components of the organs are referred to as ‘carcinomas’, whereas ‘sarcomas’ are the ones that originate from the connective tissue or the stroma of the cells and thus form rare forms of cancer. Epithelial tissues being higher than connective tissues in breast gives way to the development of carcinomas. Epithelial cells are found lining the terminal ducts and the lobules responsible for producing milk in healthy females. The majority of breast cancers fall in the category of carcinomas, and only 1% of breast cancers account for sarcomas. In case of sarcomas, the stroma cells lead to the occurrence of angiosarcoma and phyllodes tumors as these stromal cell components are rich in blood vessels and myofibroblasts [3,4].

Breast cancer develops with the certain uncontrolled cell growth taking place at the level of epithelial tissues. In situ cancer takes place at the site of epithelial tissues. The type of cancer that has not yet invaded the tissue or organs and is in the pre-invasive stage is referred to as ‘in situ carcinoma’. The growth period of these cancer cells (in situ cells) takes places in the previously normal ducts and the lobules. The potential of in situ cancer to take the form of invasive cancer is quite significant; therefore, there is an urgent need for early diagnosis and treatment to avoid the worst forms of cancer [5].

The anthracyclic agent doxorubicin (DOX)—extracted for the first time in 1970 from *Streptomyces peucetius*—is utilized as a therapeutic agent in various cancerous conditions such as lung, gastric, ovarian, breast, thyroid, multiple myeloma, sarcoma, and pediatric neoplasia [6]. DOX acts primarily on DNA via inhibiting biochemical synthesis of macromolecular structures. This leads to inhibition of enzyme topoisomerase II, and this results in the relaxation of DNA supercoiling in transcription. This anti-neoplastic agent prevents the process of replication by stabilizing enzyme topoisomerase II that leads to failure in the release of double-helix DNA. The other mechanism attributed to action of DOX is DNA and plasma membrane damage via generation of free radicals [7].

Delivery of DOX is a challenging task and conventional formulations are not effective in treating cancer. Numerous novel approaches of drug delivery for DOX [8,9] are being used to provide effective delivery of drugs with a minimum chance of resistance. One such promising and continuously evolving novel approach is nanomedicine. By definition, ‘nanomedicine’ refers to the biomedical use of materials with at least one dimension below 100 nm. Examples of nanomedicine include liposomes, nanoparticles, micelles, dendrimers [10], nanotubes, PEGylated nanoliposomes, solid lipid nanoparticles [11], hydrogels, nanostructured lipid carriers (NLCs), PLGA nanospheres, chitosan nanoparticles etc. They can be comprised of a variety of substances, such as lipids, phospholipids, polymers, proteins, inorganic sub-stances, or a mixture of these. The ionic gelation method is one of the most commonly used techniques to prepare nanoparticles, because it is a relatively easy technique to reproduce; performed under mild conditions and requires less harsh solvents; and it is also easily modified and controllable [12,13].

These novel drug delivery systems have many advantages over conventional therapy in terms of bioavailability, enhancement of solubility, stability, pharmacological activity, tissue macrophage distribution, sustained delivery, physical and chemical degradation, and protection from toxicity [14]. Moreover, these nano-drug-delivery systems can be modified into novel carriers such as pH sensitive nanoformulation [15], polymeric micro/nano carriers [16], provesicular nanogels [17], and biohybrids of bacteria and nanoparticles to achieve targeted drug delivery [18,19]. Among targeted drug delivery systems, a stable and long-acting drug carrier system is PEGylated stealth (polyethylene glycol (PEG)). Encapsulation of DOX into liposomes of less than 100 nm diameter coated with surface active agent methoxypolyethylene glycol forms a stable PEGylated-liposome encapsulated doxorubicin (PLD). This liposomal drug delivery system possesses an extensively longer plasma half-life of about 2 to 3 weeks and lower blood clearance with reduced apparent volume of distribution that is comparable with either free DOX or liposomal doxorubicin formulations [20]. A relevant work has been reported recently in which the researchers were able to design and optimize doxorubicin loaded PEGylated silver nanoparticles (AgNPs). The prepared nanoparticles exhibited a sustained release profile as compared to unloaded DOX at pH 5.0. The conclusion of the study was that the improved AgNPs-PEG may be considered an effective carrier for DOX due to its high drug loading, prolonged DOX delivery, increased synergistic cytotoxicity, and reduced side effects on healthy cells [21]. Doxorubicin loaded in mesoporous silica nanoparticles is another promising approach, reported recently, towards building stimuli-responsive nano-drug delivery systems [22]. Non-ionic surfactant vesicles (NSVs) have also been employed in loaded doxorubicin and successfully exhibited a sustained drug-release pattern at pH 5.0 [23]. Another interesting approach to develop a stimulus sensitive drug delivery system has been achieved by modifying an aqueous two-phase system (ATPS) with silica nanoparticles. MTT assay showed no toxicity of the prepared DOX-loaded drug delivery system with a good release profile at acidic pH 5.5 [24].

Such innovative drug delivery systems inspired us to develop a modified DOX-loaded system to better control the drug release with minimum toxicity. The aim of current research work was to formulate PEGylated-protamine complex (PPC) to impart the control release properties, because it has the ability to control the release of drugs that are bound with this complex. It may be useful in drug delivery system due to its cell penetration enhancing properties. Protamine in various research works has been utilized as a cell-penetrating peptide to develop the characteristics of permeation. The cationic nature of PPC will help to interact negatively charge cell membrane of tumor for easy and rapid penetration which is augmented by cell-penetrating protamine peptide. PEG will increase the retention time in the cell and provides doxorubicin to destroy cancerous cell in targeted fashion. In this study, we also investigated the binding of this complex with anticancer drug. It was hypothesized that PPC can be combined with anticancer drug, such as DOX, to control the release from the nanocomplex. Hence, nanocomplex has been generated by chemical bonding of bi-functional polyethylene glycol (PEG, MW- 2 kD) carboxylate with protamine sulphate (protamine sulphate, MW- 4.5 kD). There are specific properties attributed to this unique PPC that facilitate the tumor targeting.

## 2. Materials and Methods

### 2.1. Materials

Polyethylene glycol (PEG M.Wt 2000 D) was purchased from Daejung Chemicals & Metals Co., LTD (Siheung-si, South Korea), PEG dicarboxylate (synthesized in a laboratory through the reported method), dichloromethane (BDH Laboratory supplies), protamine sulphate (approximate M.Wt 4500 D) was a kind gift by LCPW Howards, Lahore. Tri Poly Phosphate (TPP), 1-Ethyl-3-(3-dimethylaminopropyl) carbodiimide hydrocholride (EDC-HCl), and N-hydroxysuccinimide (NHS) were purchased from Sigma-Aldrich, Taufkirchen, Germany. Doxorubicin hydrochloride was donated by Pharmedic Pharmaceuticals, Lahore, Punjab, Pakistan.

### 2.2. Methods

#### 2.2.1. Synthesis of PEG Dicarboxylate

The synthesis is carried out following a previously reported direct oxidation method [25]. 5 g of PEG2000 was dissolved in the 25 mL of water having 8 mL of concentrated sulfuric acid (96%). To this solution, we added 5 mL aqueous solution of CrO3 having mass of 0.235 g. The orange solution was subjected to stirring for 8 h at a room temperature of 20–25 °C.

25 mL of water was added into the resulting green-blue solution. Afterwards, the solution was extracted with dichloromethane (100 mL) thrice. The organic layers were combined and subjected to washing with 25 mL of water and saturated NaCl solution twice each. The organic layers were dried in petri dishes overnight and PEG-carboxylate was scratched. The solid PEG-carboxylate with 96% yield was obtained and refrigerated.

#### 2.2.2. Synthesis of PEG-Protamine Complex

**Scheme 1:**
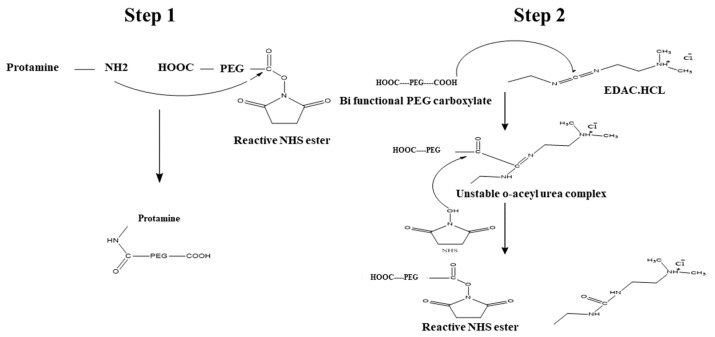
**Step 1**—Reactive NHS ester reacts with the amine functionality of protamine forming a covalent linkage between amine functional group and carboxylic acid moiety; **Step 2**—Carboxylic acid moiety of PEG carboxylate is activated by EDAC in the presence of NHS resulting in the formation of reactive NHS ester.

A new method including two steps was used to synthesize PPC [26]. Briefly, in the first step, 100 mg of protamine, 100 mg of EDAC·HCl, and 95 mg of NHS were added to a flask containing 10 mL of deionized water under constant stirring for 3 h. After completion of the reaction, a reactive NHS-ester was produced. In the second step, 100 mg of PEG 2000 was hydrated and dissolved by the addition of demineralized water to obtain a 1% solution of the polymer. Thereafter, the reactive NHS-ester was added drop-wise into the PEG solution via burette, with the pH being maintained at 5 using 10 M NaOH. The mixture was incubated at room temperature under continuous stirring for 12 h. To remove the unreacted EDAC.HCl and NHS, the polymer solutions were exhaustively dialyzed in tubing (molecular weight cut-off 1.5 kDa; dialysis tubing, cellulose membrane) five times against 5 L distilled water at 8 °C in the dark. The frozen aqueous polymer solutions of samples and controls were lyophilized then stored at 4 °C for further use (Scheme 1).

#### 2.2.3. Preparation of Blank Nanoparticles

To prepare blank nanoparticles of PPC, a solution of PPC (0.1% *w/v*) was prepared by dissolving it in deionized water with continuous stirring at 900 rpm. After that aqueous solution of TPP (0.5% *w/v*) was added slowly until opalescent suspension was formed. Stirring was continued for 45 min to reduce the size of nanoparticles. Blank nanoparticles were optimized using ranges of different concentration of polymer and TPP. Afterwards, the optimized blank nanoparticles are used to load the drug and optimized subsequently. Table 1 represents various trials conducted for optimization of blank nanoparticles.

#### 2.2.4. Preparation of Doxorubicin Loaded Nanoparticles

**Scheme 2:**
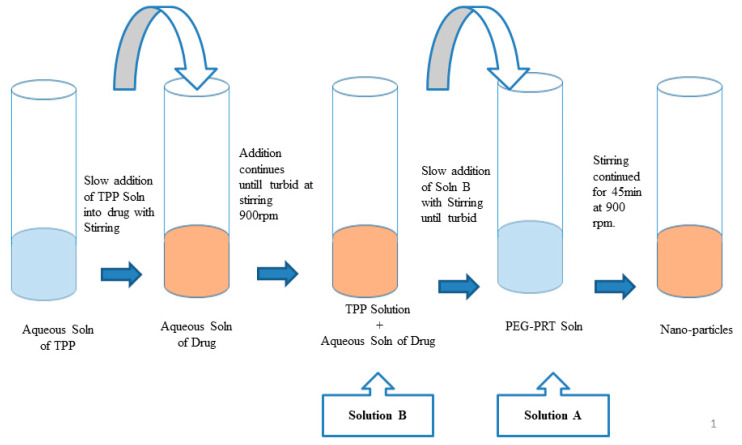
Preparation of nanocomplex by ionic gelation method.

In this experiment at step one we prepared an aqueous solution of (0.1% *w/v*) PPC (Solution A). In the second step an aqueous solution (0.5% *w/v*) of TPP was slowly injected through an insulin syringe into the DOX solution (0.1% *w/v*) until the opalescent solution (Solution B) is obtained as reported in previous literature [27]. The volume of TPP solution consumed until opalescence was 30 µL. Afterwards, in Step 3, into the Solution A (600 µL) slowly injected Solution B (400 µL) with continuous stirring at 900 rpm to obtain stable drug loaded nanoparticles. Stirring was continued for 45 min to reduce the size of nanoparticles (Scheme 2).

For optimization of formulation, the hit and trial method was used by varying the concentration and volume of TPP. Ranges of 1–2 mg or 0.1–0.2% were selected for PPC and drug respectively. TPP concentration was optimized ranging from 1–10 mg or 0.1–1%. Particle size and encapsulation efficiency were the response parameters that were checked against all formulations. Total numbers of trials were 12 for which particle size and encapsulation efficiency was determined. The best formulation was selected on the basis of smaller particle size and higher encapsulation efficiency.

After performing all experiments, selected formulation was further evaluated against various factors that were involved in the preparation of drug-loaded polymeric nanoparticles. Process parameters—such as concentrations and volume of TPP—were chosen to optimize the formulations. Twelve (12) formulations were prepared to validate preparation method.

### 2.3. Characterization of Nanoparticles

#### 2.3.1. Particle Size

The particle size, distribution, and zeta potential of the samples were determined through Zetasizer Nano ZS 90 (Malvern Instruments; Worcestershire, UK), equipped with software (version 6.34) and a He-Ne laser at a wavelength of 635 nm and static scattering angle of 90 degrees. Briefly, 10 µL of the sample was mixed with 1 mL of deionized water and vortexed for 2 min followed by analysis. Each result displayed was measured in triplicate.

#### 2.3.2. Entrapment Efficiency

For the creation of a standard calibration curve, stock solution of a drug was initially prepared by dissolving 5 mg of doxorubicin in 10 mL deionized water. By withdrawing 100, 200, 300, and 400 μL and making dilutions up to 5 mL with deionized water to obtain concentrations of 1, 2, 3, and 4 μg/mL, correspondingly, a curve was constructed. UV–visible spectrophotometer was used to perform analysis by utilizing deionized water as a control in all dilutions. A standard calibration curve was formulated to evaluate unknown concentrations of drugs in various formulations.

#### 2.3.3. Percentage Yield of Nanoparticles

Percentage yield is an important parameter for large scale manufacturing concern. To determine the percent yield, total weight of lyophilized nanoparticles was divided by total weight drug and polymer used.

(1) $$\mathrm{Percentage}\;\mathrm{yield}=\frac{\mathrm{Total}\;\mathrm{amount}\;\mathrm{of}\;\mathrm{nanoparticles}\;\mathrm{lyophilized}}{\mathrm{Amount}\;\mathrm{of}\;\mathrm{drug}\;+\;\mathrm{Pegylated}\;\mathrm{Protamine}\;\mathrm{complex}}\;\times \;100$$

#### 2.3.4. Morphology

Nanoparticles were lyophilized with help of lyophilizer (Telstar Cryodos-50, Terrassa, Spain), to obtain dried particles. A high-resolution scanning electron microscope (Tescan vega-3, model imu vp-sem, New York, NY, USA) was used to analyze the surface properties, structure, and size of the dried particles.

#### 2.3.5. Differential Scanning Calorimetery (DSC)

Differential scanning calorimetery (Hitachi High Technologies, Inc., Tokyo, Japan) analysis was performed to study the DOX physical state inside the PPC. DSC was applied at varying temperatures from 30 °C to the melting point of the drug.

#### 2.3.6. Fourier Transform Infrared (FTIR) Spectroscopy

FTIR spectrophotometer (L16000A, Perkin-Emler, Boston, MA, USA) was utilized to record the FTIR spectra. Each 10 mg sample was triturated with 100 mg of KBr (potassium bromide) and scanned between 4000 cm^−1^ and 450 cm^−1^.

#### 2.3.7. X-ray Diffraction (XRD)

X-ray diffraction analysis was performed to study the DOX physical state using a diffractometer (Rigaku Inc., Akishima, Japan). X-ray diffraction analysis was performed to study the DOX physical state inside the nanoparticles. XRD was applied at room temperature (20 kV, 5 mA). Absolute intensity was consequently recorded in a range of 5–80 against theta 2.

### 2.4. In Vitro Drug Release Studies

A dialysis membrane was used to perform in vitro release study in phosphate buffer of pH 7.4 and acetate buffer of 4.8. The formulation (4 mL) was placed in a dialysis sac to assess drug release at various time intervals. This medium was placed in a USP dissolution apparatus II containing 900 mL of buffers at 37 °C. All studies were performed in triplicate. Sampling times were 0.25, 0.5, 1, 2, 4, 6, 8, 10, 12, 18, 24, 36, and 48 h and 1 mL of sample was withdrawn. Phosphate and acetate buffers were used to replace the amount of sample withdrawn. UV–visible spectrophotometer was utilized to quantify these samples at wavelength of 480 nm. To calculate drug release, a graph was plotted between percent drug releases versus time. Several kinetic models were also applied to check the mechanism of drug release. To calculate drug release, a graph was plotted between percent drug releases versus time using Microsoft Excel. Several kinetic models—such as first order, zero order, Higuchi, Hixon–Crowel, and Korsmeyer–Peppas—were applied for evaluation of release profile.

### 2.5. Statistical Analysis

All characterization and analysis was performed in triplicate and standard deviation was also calculated. Release studies were conducted by utilizing different buffers followed by one-way analysis of variance (ANOVA).

## 3. Results

### 3.1. Synthesis of the PEG–Protamine Complex

The covalent attachment of protamine to the bi-functional PEG carboxylate was mediated by carbodiimide. The reaction is a two-step reaction. In the first step, the carboxylic acid moiety of PEG carboxylate is activated by EDAC in the presence of NHS, resulting in the formation of a reactive NHS ester. In the second step, the reactive NHS ester reacts with the amine functionality of protamine, thereby forming a covalent linkage between the amine functional group and the carboxylic acid moiety [28].

**Figure 1:**
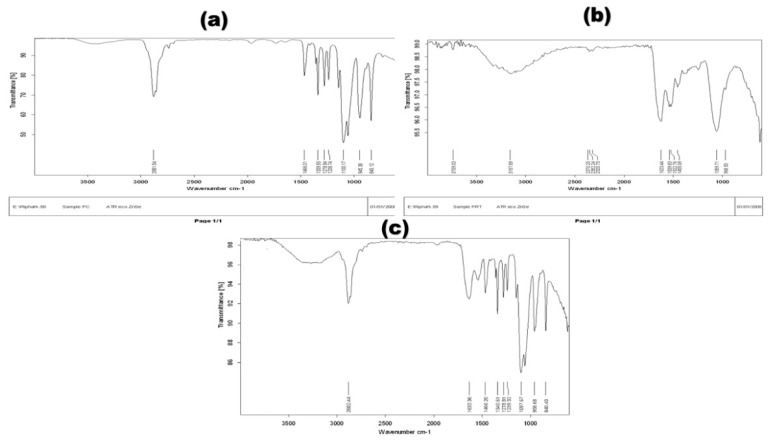
(**a**) FTIR of PEG-carboxylate, (**b**) protamine sulphate, and (**c**) PEG-protamine complex.

The reaction was confirmed by the FTIR spectra of the final complex as the appearance of the peak in the 1278 cm^−1^ confirmed the presence of amide bonds [29] as illustrated in Figure 1.

PEGylation of protamine was selected to maintain the prolonged retention time in the blood stream. By avoiding any branching side reactions, PEG was conjugated with N-terminal primary amine of protamine which was further confirmed by the FTIR spectra as already explained in the results section. The drug delivery system—along with this complex—can retain flexibility, biocompatibility, hydrophilicity, and enhanced circulation. This flexible nature of PPC allows the PEG chain to be exposed to the exterior of the nanocomplex. The stability of the formulation was confirmed after incorporating the drug inside this nanocomplex. The formulation is supposed to reduce the side effects specifically associated with existing modified drug delivery system of DOX for parenteral administration [30].

### 3.2. Optimization of Formulation

A total of 12 formulations using PPC as polymer were prepared—marked as F1 through to F12—through ionic gelation method [31]. Concentrations of Solution A and Solution B were taken as independent variables. Solution A (0.1% *w/v* of PEG-protamine complex) and Solution B (0.1% *w/v* of Doxorubicin + 0.5% *w/v* of TPP) were prepared. Concentrations of Solution A and B were varied to observe their effects on dependent variables—such as particle size, zeta potential, and polydispersity index (PDI)—as shown in Table 2. F1 formulation was selected for further testing owing to low particle size, low PDI, and high zeta potential. F1 was formulated using 600 µL of Solution A and 400 µL of Solution B.

A linear regression model was used to obtain the potential predictor in which Solution A and Solution B concentrations were taken as independent variables while particle size was taken as a dependent variable. Summary statistics indicated an excellent predictability of the model since the R2 value was 0.939 and the *p* value was <0.0001. PDI was taken as a dependent variable, summary statistics indicated an excellent predictability of the model since the R2 value was 0.855 and the *p* value was <0.0001. Moreover, when zeta potential was taken as dependent variable, summary statistics showed an R2 value of 0.267 and a *p* value of 0.247.

**Figure 2:**
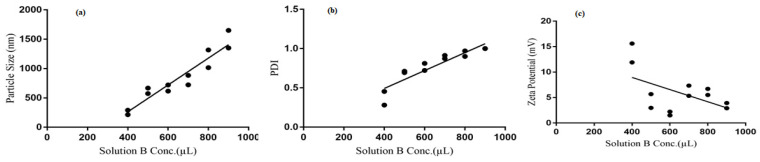
(**a**) Effect of TPP concentration on particle size; (**b**) effect of TPP concentration on PDI; (**c**) effect of TPP concentration on zeta potential.

According to the literature survey, various experiments were performed to investigate the effect of most significant parameters (TPP volume and TPP concentration) on particle size. An increase in particle size was observed when a large amount of TPP was used. This indicates the fact that the size of the nanoparticles depends upon the concentration of TPP because TPP has a tendency to coalesce at high concentrations [32]. An increase in particle size can be due to density difference between the external and internal phases or it may occur due to the reduced diffusion rate of solute molecules in the outer phase. A linear regression model for effect of polymer and TPP concentration on particle size, as shown in Table 3, predicts that increasing polymer concentration will significantly increase the particle size (β = 0.28, *p* = 0.032). The model also predicts that increasing TPP concentration significantly increases the particle size (β = 2.289, *p* < 0.0001). The effect of TPP concentration is also depicted in Figure 2a. The linear regression model for the effects of polymer and TPP concentration on PDI, as shown in Table 4, predicts that increasing polymer concentration will increase the PDI (β = 0.0001, *p* = 0.257) but the results are not significant. On the other hand, the model predicts that increasing TPP concentration significantly increases the PDI (β = 0.001, *p* = 0.001) as depicted in Figure 2b. Spearman correlation for the effect of TPP on PDI also produces non-significant results (r = −0.3534 and *p* = 0.2653). The negative r value indicates a non-significant but negative correlation showing that increasing the TPP concentration tends to decrease zeta potential as depicted in Figure 2c [1,2].

### 3.3. Particle Size, Polydispersity Index, and Zeta Potential

**Figure 3:**
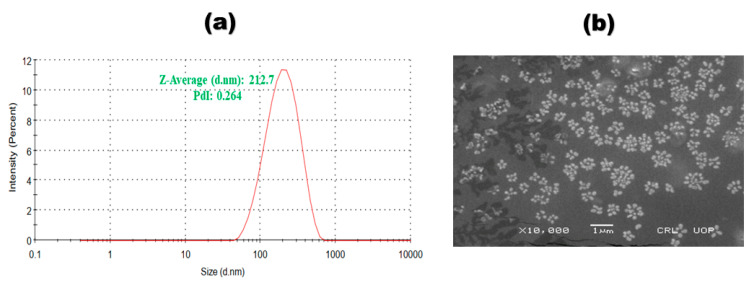
(**a**) Particle size and PDI and (**b**) SEM image of drug-loaded formulation.

The average particle size obtained was 212.7 nm with a standard deviation of 2.87 nm. PDI is the measure of size distribution of nanoparticles. Higher PDI values indicate polydisperse particles and low PDI indicates monodispersed particles. Average PDI values for optimized formulation were 0.264 with a standard deviation of 0.02. Figure 3a represents particle size and PDI of optimized formulation.

Nanocomplexes prepared by ionic gelation method displayed a smaller particle size (212.7 nm), higher polydispersity index (0.264), and zeta potential (15.2 mV), which warrants physical stability of formulation. Presence of positive charge may be due to PPC which forms a charge to the outer layer of nanoparticles. This positive charge will interact with negative charge cell membrane of tumor cells to give high permeation. PDI investigates the homogeneity of nanoparticles. When the PDI value is less than 0.1, it indicates the occurrence of a mono-dispersing system, while PDI values in range of 0.1–0.4 and more than 0.4 describe moderate dispersion and poly-dispersion respectively. Zeta potential is a significant parameter for evaluation of stability. Zeta potential having value above 30 mV indicates a thermodynamically stable system. These results are in slight contradiction with our study. Steric stabilization may be one factor due to presence of TPP. Adsorption of TPP on to the surface of nanoparticles may cause a shift in the shear plane of a particle, thus reducing the zeta potential [33].

### 3.4. Percentage Yield and Entrapment Efficiency

Percentage yield of lyophilized nanoparticles was found to be 78.41%. Entrapment efficiency was determined through UV–visible spectrophotometer, and it was found to be 99.6%.

Entrapment efficiency is a parameter of interest to deliver a drug in a higher dose more precisely at the site of action. Higher entrapment efficiency (99.6%) was found with the indirect method. Entrapment efficiency is bound to increase with increasing concentration of TPP, because it acts as a polyanionic cross linker agent [34]. Use of PPC results in enormous attachment of drug molecules with those of the polymer with the aid of TPP, resulting in the entrapment of drug molecules [35].

### 3.5. Scanning Electron Microscopy (SEM)

Scanning electron microscopy (SEM) was utilized to determine the morphology of blank and drug-loaded polymer complexes. Figure 3b exhibits SEM images at different intensifications. The results confirm spherical complexes in the nano-scale range.

### 3.6. Fourier Transform Infrared Spectroscopy (FTIR)

FTIR analysis was used to study the chemical stability of drug in PPC. Spectra of DOX exhibited characteristic peaks at 3253 cm^−1^ due to stretching vibration of OH groups. The peak at 2120 cm^−1^ is due to stretching vibration of the CH group. The peak at 1636 cm^−1^ is due to the stretching vibration of the amide bond [36].

**Figure 4:**
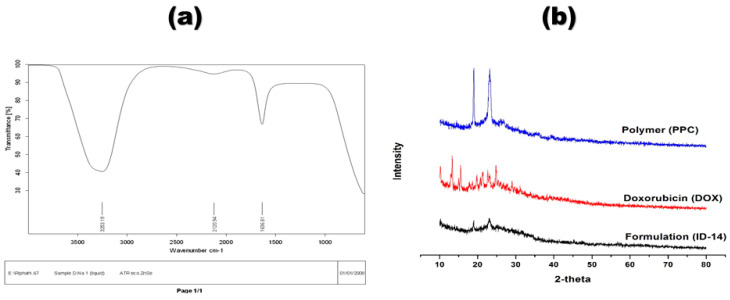
(**a**) FTIR spectrum and (**b**) XRD analysis of optimized formulation.

FTIR was used to conduct the compatibility studies between DOX and excipients (Figure 4a). FTIR analysis confirmed that there was no chemical or electrostatic interaction between ingredients of nanoparticulate formulation. Absence of any new peak or functional group also exhibits absence of any interaction among constituents. Other studies also reported a similar conclusion from FTIR analysis [37].

### 3.7. X-ray Diffraction (XRD)

X-ray diffraction analysis explains the crystalline nature of the drug as shown in Figure 4. Low intensity of peaks in the formulation indicates that the drug is successfully incorporated in the nano-carrier. Figure 4b shows that all peaks appear in the range of 10–30.

DSC and XRD analysis concluded that the drug was present in an amorphous form within the PPC. The decrease in crystallinity might be due to absorption of the cross linker on the surface of nanoparticles. The results are in harmony with previous studies [38].

### 3.8. Differential Scanning Calorimetery (DSC)

**Figure 5:**
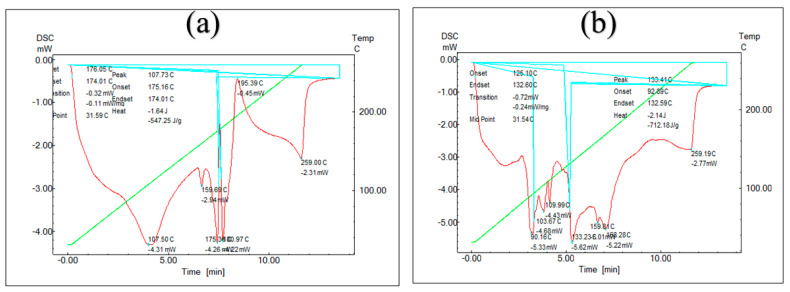
(**a**) DSC analysis of doxorubicin and (**b**) optimized formulation.

Differential scanning calorimetery analysis was performed to assess the crystalline nature of formulation. DSC analysis confirmed the amorphous nature of nanoparticles, which means that the drug was successfully incorporated into the PPC (Figure 5).

### 3.9. In Vitro Release

**Figure 6:**
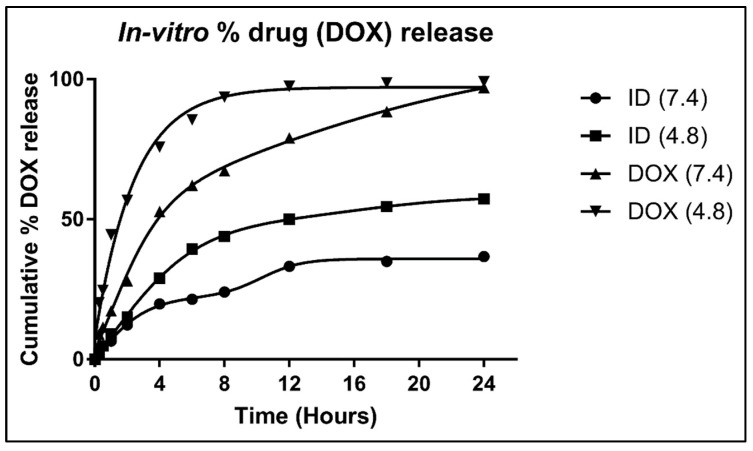
In vitro release profiles at pH 7.4 and pH 4.8 following Korsmeyer–Peppas model.

Dialysis membrane method was adopted to evaluate drug release from PPC at pH 7.4 and 4.8. Figure 6 illustrates that at pH 4.8 nearly 45% and at pH 7.4 nearly 36% of all entrapped drug was released in 13 h. At same pH values, nearly 20% and 25% was released in first 4 h respectively. While 38% and 60% of the drug was released from the polymer complex, enabling a more sustained release pattern in comparison to the drug solution which was 100% released in 24 h at an identical pH (Figure 6).

By applying various release models—such as zero order, first order, Baker Lonsdale, Hixon–Crowel, and Korsmeyer–Peppas—mechanism of drug release was determined. Rate constant and R2 values indicate that release from PPC follows the Korsmeyer–Peppas model. The data describe the results of the most suitable model. The R2 values are 0.9726 and 0.9517 at pH 7.4 and 4.8, respectively. It also illustrates that release from PPC will follow the Korsmeyer–Peppas model. The best fit values of ‘n’ at both pHs are 0.466 and 0.480, which lies in between 0.45 and 0.89, indicating that drug release will follow anomalous non-Fickian release.

## 4. Conclusions

PPC of DOX was formulated through the ionic gelation method. Self-assembled nanocomplexes were formed by the simple mixing of polycationic PEG–protamine with polyanionic TPP without further chemical modification. The PPC loaded with DOX exhibited smaller particle size and much improved entrapment efficiency. FTIR spectra revealed no interaction among excipients. Furthermore, DSC and XRD analysis confirmed the amorphous nature of formulation. The in vitro release data indicate increased and sustained drug release at pH 4.8, which is intracellular pH of breast cancer cells. Therefore, it can be concluded that the PEG–protamine nanocarrier might act as a sustained delivery system for the enhanced therapeutic efficacy of loaded DOX for targeting tumor tissues. These findings suggest that PPC loaded with DOX can be used as a surrogate for traditionally available products.

## Figures and Tables

**Table 1:** Optimization parameters for development of blank nanoparticles.

Formulation Name	Conc. of Polymer	Conc. of TPP	Volume of TPP Used	Stirring Speed	Stirring Time
**F1**	0.1%	0.8%	20 µL	900	45 min
**F2**	0.1%	0.5%	15 µL	900	45 min
**F3**	0.1%	0.6%	10 µL	900	45 min
**F4**	0.1%	0.7%	10 µL	900	45 min
**F5**	0.1%	1.0%	10 µL	900	45 min
**F6**	0.2%	0.5%	40 µL	900	45 min

**Table 2:** Effect of polymer and TPP concentration on particle size, zeta potential, and PDI.

Formulation Name	Solution A (µL) (Polymer)	Solution B (µL) (Drug + TPP)	Particle Size	Zeta Potential	PDI
F1	600	400	212.7 ± 2.87	15.2 ± 2.62	0.26 ± 0.02
F2	600	500	574.6 ± 16.1	2.98 ± 0.15	0.694 ± 0.12
F3	600	600	616.4 ± 12.8	1.51 ± 0.3	0.722 ± 0.09
F4	600	700	723 ± 9.3	7.34 ± 0.9	0.873 ± 0.18
F5	600	800	1015 ± 21.7	5.51 ± 1.1	0.90 ± 0.03
F6	600	900	1350 ± 33.5	2.91 ± 1.1	0.96 ± 0.02
F7	1200	400	290.3 ± 25.1	11.9 ± 3.3	0.454 ± 0.36
F8	1200	500	667.6 ± 32.3	5.68 ± 0.7	0.712 ± 0.2
F9	1200	600	721.1 ± 32.8	2.22 ± 0.1	0.812 ± 0.03
F10	1200	700	884.2 ± 44.1	5.34 ± 1.2	0.913 ± 0.01
F11	1200	800	1317.7 ± 27.9	6.71 ± 1.5	0.971 ± 0.01
F12	1200	900	1650.1 ± 55.7	3.93 ± 0.1	0.98 ± 0.02

**Table 3:** Linear regression analysis for effect of independent variables on particle size.

Independent Variables	β Coefficient	*p*-Value
Solution A	0.288	0.032
Solution B	2.289	<0.0001

**Table 4:** Linear regression analysis for effect of independent variables on PDI.

Independent Variables	β Coefficient	*p*-Value
Solution A	0.0001	0.257
Solution B	0.001	<0.001

## Data Availability

Not applicable.
